# Subfamily Coleoscirinae (Acari: Trombidiformes: Cunaxidae), with description of one new species from Pakistan

**DOI:** 10.1093/jis/14.1.82

**Published:** 2014-07-07

**Authors:** Muhammad Hamid Bashir, Muhammad Afzal, Muhammad Ashfaq, Shaukat Ali, Muhammad Kamran, Sabyan Faris Honey

**Affiliations:** 1 Department of Agri. Entomology, University of Agriculture, Faisalabad, Pakistan; 2 University College of Agriculture, University of Sargodha, Sargodha, Pakistan; 3 College of Natural Resource and Environment, South China Agricultural University, China

**Keywords:** Neoscirulini, Prostigmata

## Abstract

The Coleoscirinae (Acari: Trombidiformes: Cunaxidae) from Pakistan are summarized in this paper. Two species of *Scutascirus* Den Heyer ( *S. pirgus* Chaudhri and Akbar and *S* . *tactus* Chaudhri and Akbar), ten species of *Coleoscirus* Berlese ( *C. baptos* (Chaudhri and Akbar), *C. carex* (Inayatullah and Shahid), *C. carnus* Muhammad and Chaudhri, *C. comis* Muhammad and Chaudhri, *C. disparis* Muhammad and Chaudhri, *C* . *irroratus* Muhammad and Chaudhri, *C. mardi* (Inatullah and Shahid), *C. raviensis* Afzal, Ashfaq and Khan, *C* . *tobaensis* Bashir, Afzal, Ashfaq, and Khan, and *C* . *trudus* Bashir, Afzal and Akbar), and three species of *Pseudobonzia* Smiley ( *P. ashfaqi* Bashir, Afzal and Akbar, *P. numida* Chaudhri and Akbar, and *P* . *parilus* Chaudhri) have been previously reported. One new species of *Pseudobonzia* , *Pseudobonzia bakeri***sp. n.** , is herein described and illustrated. A key to the genera of the subfamily and keys to the species in each genus are given to incorporate the new species from Pakistan. Distribution records of all known species in Pakistan are also given.

## Introduction

Cunaxidae (Acari: Trombidiformes) are important predatory mites. They have been reported to feed on other small arthropods, including harmful mites and soft bodied insects (Nesbsitt 1946; Lord 1949 ; Schruft 1971 ; Kuznetzov and Sizova 1978 ; Youssef et al. 1980 ; Smiley 1992 ) and nematodes ( Walter and Kaplan 1991 ). Cunaxidae occupy diverse habitats and have been reported from all parts of the world ( Walter and Kaplan 1991 ; Swift 1996 ; Walter 1999 ; Arbabi et al *.* 2002 ; Tagore and Putatunda 2003 ; de-Oliveira and Daemon 2003 ; Tagore and Putatunda 2003 ; Sionti 2003 ; Sergeyenko 2005 , 2006, 2009, 2011; Mejia-Recamier and Palacios-Vargas 2007; Corpuz-Raros 2007 ; Kaluz 2009 ; Den Heyer 2011 ; Skvarla et al. 2011 ; Skvarla and Dowling 2012 ).

Coleoscirinae was erected by Den Heyer (1979). It encompasses two tribes (Cole-oscirini and Neoscirulini) and six genera *(Neoscirula* Den Heyer, *Pseudobonzia* Smiley *, Coleobonzia* Den Heyer & Castro, *Coleoscirus* Berlese, *Orangescirula* Bu & Li, and *Scutascirus* Den Heyer). Ninety-one species have thus far been described in this subfamily ( Den Heyer 2011 ). *Neoscirula,* which Smiley (1992) transferred to Bonzinae based on characteristics of the hypostomal setae, has recently been placed back into Coleoscirinae ( Den Heyer 2011 ).

Berlese (1916) erected the genus *Coleoscirus* and included two species, *C. halacaroides* and *C. corniculatus,* in it. Den Heyer (1978) synonymized *Coleoscirus corniculatus* with *Scirus curtipalpis.*Berlese (1916) designated *Coleoscirus halacaroides* as the type species of genus *Coleoscirus,* in spite of an earlier described species, *Coleoscirus curtipalpis* ( Berlese 1888 ). Ewing (1917) described a new species *Scirus* (= *Coleoscirus) simplex* from the U.S.A.

Thor and Willmann (1941) redescribed and provided drawings of *Coleoscirus simplex, C brevicornis, C curtipalpis, C halacaroides,* and *C curtipalpis* (as *C. corniculatus).*Baker and Hoffmann (1948) redescribed and provided the drawings of *C curtipalpis, C brevicornis,* and *C simplex.* They also described and gave drawings of a new species, *Cunaxa mexicana* (= *Coleoscirus mexicanus*Baker & Hoffmann, 1948 ) from Mexico and the U.S.A.

Smiley (1975) provided a new genus name, *Pseudocunaxa,* for species related to and including *Coleoscirus simplex..* This new genus name is a synonym of *Coleoscirus*Berlese, 1916 ( Den Heyer 1980a ). Den Heyer (1979) described two new species, *C tuberculatus* and *C magdalenae,* from Africa and gave a key for these two species. Den Heyer (1980a) described three new species, *Coleoscirus coatesi, C buartus,* and *C breslauensis,* and prepared a comprehensive key. Den Heyer (1980b) included the genus *Coleoscirus* in the subfamily Coleoscirinae, in his new tribe Col-eoscirini, along with the genus *Scutascirus.* He mentioned the genus *Pseudocunaxa*Smiley, 1975 as synonym of genus *Coleoscirus.*Tseng (1980) erected a new genus, *Lapicunaxa,* with *Lapicunaxa horidula* as its type species.

Den Heyer (1979) synonymized *Pseudocunaxa* with *Coleoscirus.*Chaudhri and Akbar (1985) and Inayatullah and Shahid (1993) apparently missed that publication or did not agree with it and described species in the genus *Pseudocunaxa.* In 1992, Smiley gave the new classification system for Cunaxidae family, as he synonymized *Pseudocunaxa* and *Lapicunaxa* with *Coleoscirus* and described 11 species of this genus, including the species of Chaudhri and Akbar (1985) . Later, Muhammad and Chaudhri (1992a, b) and Bashir et al. (2006, 2008) contributed to the fauna of this genus from Pakistan.

The genus *Pseudobonzia* was erected by Smiley (1975) . He designated *Cunaxa reticulata* Heryford as its type species. Den Heyer (1977) described six new species from the Ethiopian region and placed this genus in the subfamily Coleoscirinae ( Den Heyer 1980b ). Later, Den Heyer (1980a) , Luxton (1982) , Liang (1984) , Sepasgosarian (1984) , Chaudhri and Akbar (1985) , Michocka (1987), Smiley (1992) , and Bashir et al. (2008) made significant contributions to the fauna of this genus worldwide.

The genus *Scutascirus* was erected by Den Heyer (1976) . He designated *Scutascirus pol-yscutosus* as its type species. Den Heyer (1979, 1980a) , Sepasgosarian (1984) , Chaudhri and Akbar (1985) , Smiley (1992) , and Lin et al. (2001) have contributed to the knowledge of Cunaxidae.

## Materials and Methods

Sieve collection was used for field collecting. The plant parts, such as leaves, twigs, and inflorescences, were beaten on a sieve held over a white piece of paper. Cunaxids were sorted with the help of a magnifying lens and stored in vials containing 70% alcohol and few drops of glycerin. Materials such as soil and leaf debris that could not be processed in the field were processed in Berlese funnels for at least 24 hours. They were subsequently sorted under a binocular microscope and preserved in 70% ethanol. The specimens were mounted permanently on glass slides using Hoyer’s medium and identified using a phase contrast microscope. Illustrations were prepared by using an ocular grid. The identification of the species was done with the help of existing keys and literature. The setal nomenclature of Kethley (1990) has been adopted. All the measurements (in um) and ranges are given in the description. The following abbreviations are used in this manuscript:

asl: attenuate solenidion

bsl: blunt ended solenidion

sts: simple tactile setae

T: trichobothrium

Peo: cunaxid peg organ on tarsi I

### Nomenclature

This publication and the nomenclature it contains have been registered in Zoobank. The LSID number is:

urn:lsid:zoobank.org :pub:13C6436F-7557-439D-B4A1-A7C8F07D3530

It can be found online by inserting the LSID number after www.zoobank.org/

## Results and Discussion

### Key to genera of the subfamily Coleoscirinae ( Smiley, 1992 )

1) Ventral idiosoma with sub triangular plate adjacent to ventrolateral coxal and genital plates *-----------------Scutascirus* Den Heyer Ventral idiosoma without sub triangular plate adjacent to ventrolateral coxal and genital plates -----------------------------------2

2) Female with sternal and ventrolateral plates. Dorsum with a single shield extending from propodosoma into hysterosomal region------------------------- *Coleoscirus* Berlese - Female without sternal and ventrolateral plates. Dorsum with a single shield confined to propodosomal region --------------------------------------------- *Pseudobonzia* Smiley

### Key to species of the genus *Scutascirus* known from Pakistan

1) Gnathosoma with striations at base, palp telofemur with 1 seta ---------------------------- *tactus* Chaudhri & Akbar

2) Gnathosoma with reticulations at base, palp telofemur with 1 seta and 1 spine ---------------------- *pigrus* Chaudhri & Akbar

***Scutascirus pigrus*** Chaudhri & Akbar *Scutascirus pigrus* Chaudhri & Akbar, 1985: 231

Known distribution: Pakistan: Faisalabad Known source: Rawan ( *Vigna sinensis* )

***Scutascirus tactus*** Chaudhri & Akbar *Scutascirus tactus* Chaudhri & Akbar, 1985: 229

Known distribution: Pakistan: Gujranwala Known source: Jute ( *Corchorus capsularis* )

### Key to species of the genus *Coleoscirus* known from Pakistan

1) Dorsal shield with 4 pairs of setae in hysterosomal region --------------------- 2

- Dorsal shield with more than 4 pairs of setae in hysterosomal region ---------------------------- 4

2) Palp tibiotarsus with large spur on inner medial surface; genital shield with 4 pairs of simple setae ------------------------------------- *trudus* Bashir & Afzal - Palp tibiotarsus with small tubercle on inner medial surface; genital shield with 3 pairs of simple setae ------------------------------ 3

3) Genu IV with 5 setae; tibia I with 7 setae ------------------------- *carex* (Inayatullah & Shahid) - Genu IV with 6 setae; tibia I with 6 setae ------------------------- *mardi* (Inayatullah & Shahid)

4) Dorsal shield with 6 pairs of simple setae in hysterosomal region -------------------------5 - Dorsal shield with 5 pairs of simple setae in hysterosomal region -------------------------7

5) Venter with 6 pairs of simple setae between lateral plates and distal part of body in addition to setae of anal and genital region-------------------------- *raviensis* Bashir, Afzal, Ashfaq & Khan Venter with less than 6 pairs of simple setae between lateral plates and distal part of body in addition to setae of anal and genital region -------------------------6

6) Leg genu IV with 6 setae; venter with 4 pairs of simple setae between lateral plates and genital region ------------------------- *tobaensis* Bashir, Afzal, Ashfaq & Khan - Leg genu IV with 7 setae; Venter with 5 pairs of simple setae between lateral plates and genital region ------------------------- *carnus* Muhammad & Chaudhri

7) Coxa II with 3 setae-------------------------8

- Coxa II with 2 setae------------------------- *baptos* (Chaudhri & Akbar)

8) Basifemur III with 4 setae-------------------------9 - Basifemur III with 5 setae -------------------------10

9) Genu I with 8 setae; telofemur I with 4 setae ------------------------- *simplex* (Ewing)

- Genu I with 9 setae; telofemora I with 5 setae ------------------------- *irroratus* Muhammad & Chaudhri

10) ara anal seta present------------------------- *comis* Muhammad & Chaudhri - Para anal seta absent -------------------------11

11) Genu I-IV with 8-7-6-6 setae------------------------- *kayfayati* (Inayatullah & Shahid) - Genu I-IV with 9-8-6-7 setae ------------------------- *disparis* Muhammad & Chaudhri

****Coleoscirus baptos**** (Chaudhri & Akbar) *Pseudocunaxa baptos* Chaudhri & Akbar, 1985: 223. Known distribution: Pakistan: Charrapani (Murree) Known source: Pine *(Pinus* spp.)

*Coleoscirus carex* (Inayatullah & Shahid)

*Pseudocunaxa carex* Inayatullah & Shahid,

1993: 318

Known distribution: Pakistan: Peshawar

Known source: Rotten leaves

*Coleoscirus carnus* Muhammad & Chaudhri

*Coleoscirus carnus* Muhammad & Chaudhri, 1992: 309

Known distribution: Pakistan: Peshawar

Known source: Pear *(Pyrus communis)*

*Coleoscirus comis* Muhammad & Chaudhri

*Coleoscirus comis* Muhammad & Chaudhri,

1992: 99

Known distribution: Pakistan: Faisalabad

Known source: Stored wheat

*Coleoscirus disparis* Muhammad & Chaudhri

*Coleoscirus disparis* Muhammad & Chaudhri, 1992: 310

Known distribution: Pakistan: Karachi

Known source: Stored rice ( *Oryza sativa* )

*Coleoscirus irroratus* Muhammad & Chaudhri

*Coleoscirus irroratus* Muhammad & Chaudhri, 1992: 99

Known distribution: Pakistan: Shahkot, Gujranwala, Faisalabad

Known source: Wheat *(Triticum aestivum),* sugarcane, debris

*Coleoscirus kifayati* (Inayatullah & Shahid)

*Pseudocunaxa kifayati* Inayatullah & Shahid,

1993: 315

Known distribution: Pakistan: Peshawar

Known source: Banana *(Musa paradisiaca)*

*Coleoscirus mardi* (Inatullah & Shahid)

*Pseudocunaxa mardi* Inatullah & Shahid,

1993: 316

Known distribution: Pakistan: Mingora (Swat)

Known source: Rice ( *Oryza sativa* )

*Coleoscirus raviensis* Bashir, Afzal, Ashfaq & Khan

*Coleoscirus raviensis* Bashir, Afzal, Ashfaq & Khan, 2008: 453

Known distribution: Pakistan **:** Lahore,

Kasoor, Sialkot, Bahawalpur

Known source: Plant debris

*Coleoscirus simplex* Ewing

*Scirus* simplex Ewing, 1917 : 150

*Cunaxa* simplex (Ewing), Thor & Willmann,

1941: 172; Baker & Hoffmann, 1948: 240;

Muma, 1960: 324; Shiba, 1978: 114

*Pseudocunaxa* simplex Smiley, 1975 : 241;

Chaudhri, 1977 :43; 1985:223; Inayatullah & Shahid, 1993: 315

*Coleoscirus* simplex (Ewing), den Heyer,

1979c: 524; 1980d: 105; Sepasgosaran, 1984:

143

Known distribution: Pakistan: Faisalabad, Multan, Khanewal, Layyah, Lodhran, Toba Tek Singh, Lahore, Okara, Sahiwal, Gujranwala,

Known source: Plant debris, cucumber, tomato, brinjal, citrus

*Coleoscirus tobaensis* Bashir, Afzal, Ashfaq & Khan

*Coleoscirus tobaensis* Bashir, Afzal, Ashfaq & Khan, 2008: 455

Known distribution: Pakistan: Faisalabad,

T.T. Singh, Chakwal, Sialkot

Known source: Plant debris

*Coleoscirus trudus* Bashir, Afzal & Khan

*Coleoscirus trudus* Bashir, Afzal & Khan, 2006: 74

Known distribution: Pakistan: Faisalabad,

Muzzafarghar, Toba Tek Singh, Kasoor, D.G.

Khan

Known source: Leaf debris

**Key to species of genus** *Pseudobonzia* **known from Pakistan**

1) Palp tibiotarsus with thick spine-like seta-------------------------2

- Palp tibiotarsus without thick spine-like seta ------------------------- *bakeri,***sp. n.**

2) Ventral hysterosoma with 5 pairs simple setae between coxae II and distal part of the body excluding setae of anal and genital region ------------------------- *parilus* Chaudhri

- Ventral hysterosoma with more than 5 pairs simple setae between coxae II and distal part of the body excluding setae of anal and genital region -------------------------3

3) Venter with 7 pairs simple setae between coxae II and distal part of the body in addition to setae of anal and genital region; coxa IV with 2 setae ------------------------- *numida* Chaudhri & Akbar - Venter with 6 pairs simple setae between coxae II and distal part of the body in addition to setae of anal and genital region; coxa IV with 3 setae ------------------------- *ashfaqi* Bashir, Afzal & Akbar

*Pseudobonzia ashfaqi* Bashir, Afzal & Akbar

*Pseudobonzia ashfaqi* Bashir, Afzal and Akbar, 2008: 77

Known distribution: Pakistan: Faisalabad

Known source: Plant debris

*Pseudobonzia numida* Chaudhri & Akbar

*Pseudobonzia numida* Chaudhri & Akbar, 1985: 220

Known distribution: Pakistan: Hassanabdal

Known source: Fungus

*Pseudobonzia parilus* Chaudhri

*Pseudobonzia parilus* Chaudhri, 1977 : 45

Known distribution: Pakistan: Sialkot

Known source: Chili peppers ( *Capsicum frutescens* )

*Pseudobonzia bakeri,* Bashir, Afzal, Ashfaq,

Raza & Kamran, **sp. n.**

(Figures 1–4)

### Female

#### Gnathosoma.

**Figure 1.**
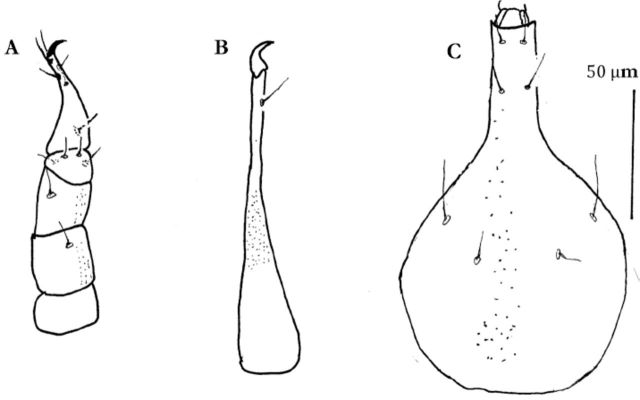
*Pseudobonzia bakeri* , **sp. n.** A: Hypostome; B: Palp; C: Chelicera. High quality figures are available online.

Gnathosoma 140 long and 80 wide. Hypostome sub rectangular, cone shaped distally; with 4 pairs hypognathal setae (hg _1_ –hg _4_ ) ( Figure 1A ). Palp 5 segmented, measuring 110. Chaetotaxy of palp as follows: trochanter none; basifemur with one simple seta; telofemur with one simple seta; genu with 4 simple setae; tibiotarsus terminating in a claw, with 6 simple setae ( Figure 1B ).

Chelicerae 135 long, terminating in a claw, with one simple dorsomedial seta, dorsal and ventral sides with papillae ( Figure 1C ).

#### Dorsum.

Body 370 long (without gnathosoma) and 270 wide. Propodosoma with a weakly sclerotized sub rectangular shield bearing randomly placed different sized papillae. Propodosomal shield with sensillae *vi* and *sce* measuring 100 and 115, respectively, and propodosomal setae *ve* measuring 17, *sci* measuring 11.

**Figure 2.**
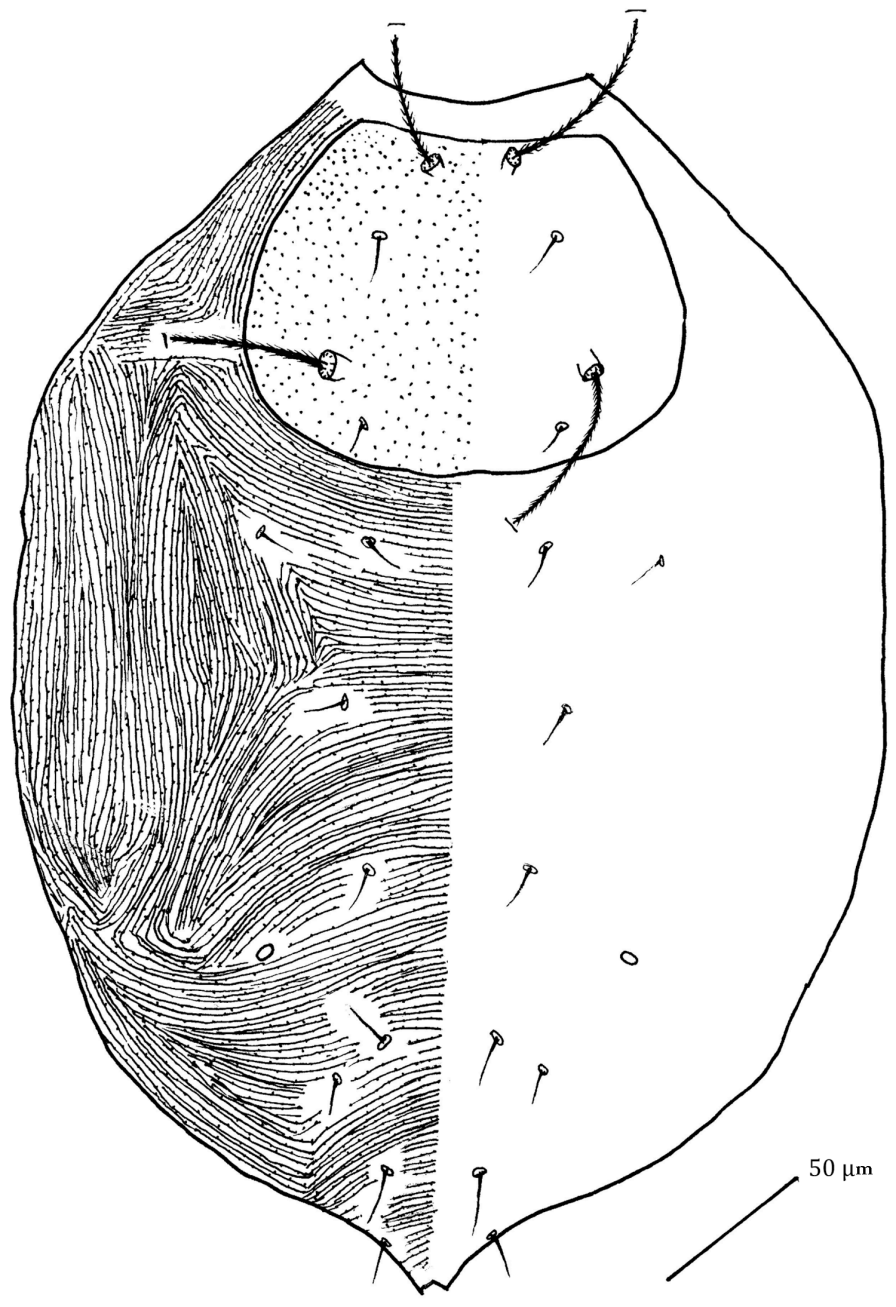
*Pseudobonzia bakeri* , **sp. n.** Dorsal side. High quality figures are available online.

Hysterosoma separated from propodosoma by papillae bearing striae. Hysterosoma with setae *c1* , *c2* , *d1* , *e1* , *f1* , *f2* , *h1* , and *h2* measuring 11, 11, 11, 11, 13, 12, 17, and 17, respectively. Hysterosoma with one pair of cupules, *im,* on integument anterior to setae *f1* ( Figure 2 ).

#### Venter.

**Figure 3.**
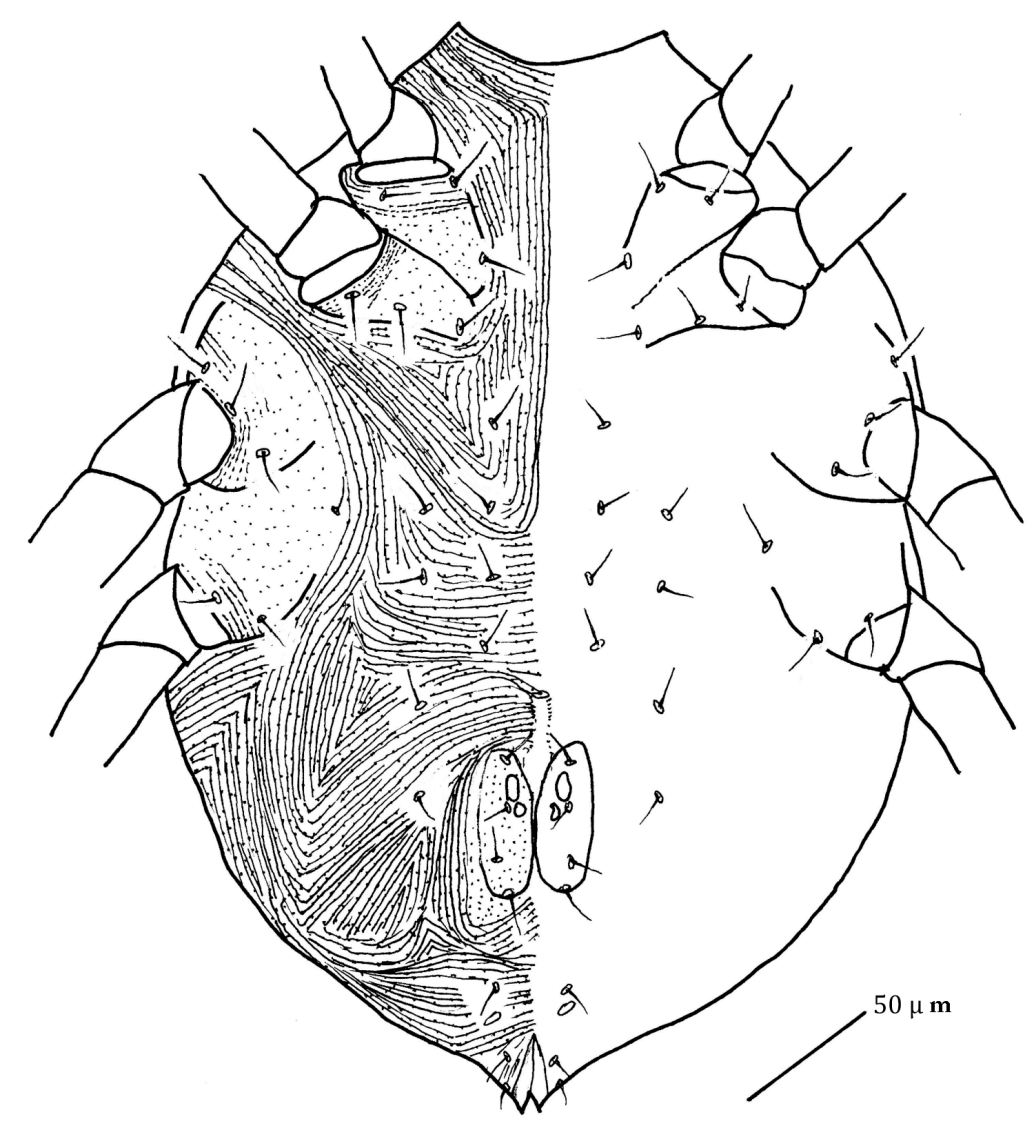
*Pseudobonzia bakeri* , **sp. n.** Ventral side. High quality figures are available online.

Venter with papillate striations. Coxae I–II contiguous, connected by small lateral apodemes; coxae III–IV contiguous, broader than coxae I–II. Ventral hysterosoma with 1 pairs propodogastral simple setae and 7 pairs hysterogastral setae in addition to setae of anal and genital region. Genital shield with two valves bearing papillae. Each valve with 4 genital setae *(g _1_* – *g4)* longitudinal aligned and 2 genital suckers. Two pairs of anal setae *(a)* and one pair of paranal setae *(pa)* present. One pair minute pores near anal shield ( Figure 3 ).

#### Legs.

**Figure 4.**
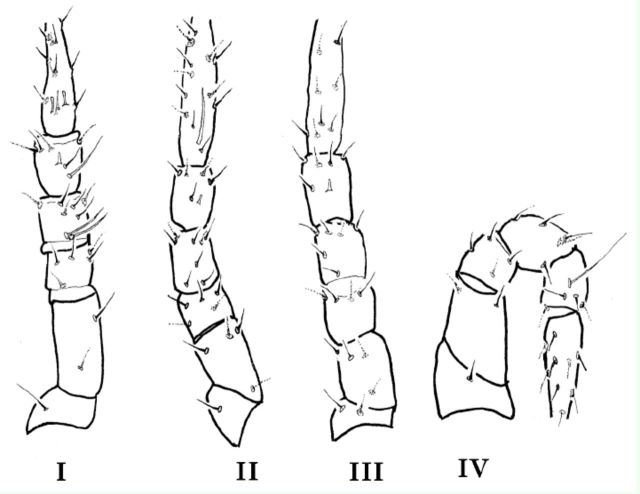
*Pseudobonzia bakeri,* **sp. n.** Legs I–IV. High quality figures are available online.

All legs pitted with papillae and blunt ended. Legs I-IV measuring (from trochanter base to the tip of tarsus) 193, 185, 185, and 187 respectively. Chaetotaxy of legs I-IV as follows: Coxae 3-3-3-3 *sts;* trochanters 1-1-2-1 *sts;* basifemora 2-4-4-2 *sts;* telofemora 5-5-4-4 *sts;* genua 8 (3 *asl +* 5 *sts)-8-6-5 sts;* tibiae 7 (1 *asl +* 1 *bsl +* 5 *sts)-6* (1 *bsl +* 5 *sts)-6* (1 *bsl +* 5 *sts)-5* (1 *T* + 4 *sts)* and tarsi 25 (3 *asl +* 1 *peo+ 2 bsl* + 19 *sts)-25* (1 *asl +* 24 *sts)-18-* 16 *sts* ( Figure 4 ).

#### Male

Unknown.

### Type material.

Holotype female, collected in Lahore from leaf debris on 28 August 2004 (Hamid) and deposited in the Acarology Research Laboratory, Department of Agri. Entomology, University of Agriculture, Pakistan.

### Etymology.

The species epithet is in reference to Dr. Edward W. Baker, Research Entomologist (Acarology), Systematic Entomology Laboratory, U.S. Department of Agriculture, Beltsville, Maryland, USA, for his outstanding contribution to the field of Acarology.

### Remarks.

This new species, *Pseudobonzia bakeri***sp. n.** , is very similar to *P. summersi* Smiley but can be separated by the following characters: 1) Ventral hysterosoma with 5 pairs hysterogastral setae in *P. summersi* , compared to 7 pairs in *P. bakeri***sp. n.** ; 2) Chaetotaxy of legs I–IV in *P. summersi* is: basifemora 4-6-4-2, telofemora 5-5-4-3, genua 8-7-6-6, tibiae 7-6-6-5, and tarsi 18-19-20-18, whereas in *P. bakeri***sp. n.** the chaetotaxy is: basifemora 2-4-4-2, telofemora 5-5-4-4, genua 8-8-6-5, tibiae 7-6-6-5, and tarsi 25-25-18-16.

*Pseudobonzia bakeri* **sp. n.** can be separated from *P. clathratus* (Shiba) by the following characters: 1) Palp tibiotarsus with 5 simple setae in *P. clathratus* , whereas with 6 simple setae in *P. bakeri***sp. n.** ; 2) Venter with 6 pairs of hysterogastral setae in *P. clathratus* , compared to 7 pairs in *P. bakeri***sp. n.** ; 3) Chaetotaxy of legs I–IV in *P. clathratus* is: basifemora 3-3-2-1, telofemora 6-5-4-3, genua 9-6-6-6, tibiae 8-6-6-5, and tarsi 27-21-18-14, whereas in *P. bakeri***sp. n.** the chaetotaxy is: basifemora 2-4-4-1, telofemora 4-5-4-4, genua 8-8-5-5, tibiae 7-6-5-5, and tarsi 24-25-18-16.

This new species can also be compared with *P. delfinadobakerae* Smiley, however, they can be separated by the combination of the following features: 1) Palp tibiotarsus with a thick spine-like seta in *P. delfinadobakerae* , whereas it is absent in *P. bakeri***sp. n.** ; 2) Venteral hysterosoma with 6 pairs of hysterogastral setae in *P. delfinadobakerae* , compared to 7 pairs in *P. bakeri***sp. n.** ; 3) Chaetotaxy of legs I–IV in *P. delfinadobakerae* is: basifemora 3-3-2-1, telofemora 6-5-4-3, genua 9-7-6-6, tibiae 7-6-6-5, and tarsi 26-21-18-14, whereas in *P. bakeri***sp. n.** the chaetotaxy is: basifemora 2-4-4-1, telofemora 4-5-4-4, genua 8-8-5-5, tibiae 7-6-5-5, and tarsi 24-25-18-16; 4) Genital shield and coxae are papillate in *P. bakeri***sp. n.** , whereas they are reticulated in *P. delfinadobakerae.*
